# Quality Changes in Chicken Meat Marinated With Antioxidant‐Rich Fruit and Vegetable Juices

**DOI:** 10.1002/fsn3.70135

**Published:** 2025-04-02

**Authors:** İlkay Çelik, Eda Alagöz, Hülya Şen Arslan, Cemalettin Sarıçoban

**Affiliations:** ^1^ Ege University, Departman of Food Engineering İzmir Turkey; ^2^ Selçuk University, Departman of Food Engineering Konya Turkey; ^3^ Karamanoğlu Mehmetbey University, Departman of Food Engineering Karaman Turkey

**Keywords:** black carrot, chicken breast meat, pomegranate, red beet

## Abstract

This study investigated the effects of marination with antioxidant‐rich fruit juices—pomegranate, black carrot, and red beet—on the textural, physicochemical, technological, and sensory properties of chicken breast meat. Key parameters, including color, pH, water‐holding capacity (WHC), marinade absorption, cooking loss (CL), textural properties, TBARS (thiobarbituric acid reactive substances), and sensory attributes, were evaluated. Among the tested juices, pomegranate had the highest acidity. Black carrot contained the highest total phenolic content (TPC) but showed the lowest ABTS and DPPH radical scavenging activity (*p* < 0.05). Marination resulted in a reduction in pH, with the lowest values observed in pomegranate‐marinated samples. Although marination influenced WHC, the changes were not statistically significant (*p* > 0.05). CL values were significantly reduced in marinated samples (*p* < 0.05). Marination also affected color, with red beet increasing the b* value and black carrot decreasing it (*p* < 0.05). Textural properties, such as hardness, significantly increased with pomegranate and red beet juices (*p* < 0.05), while other textural attributes remained unaffected (*p* > 0.05). Sensory evaluation revealed no significant differences in flavor and texture, although color was notably influenced by the marination process (*p* < 0.05). Control and RB gave the highest values in terms of general acceptability. These findings suggest that marination with pomegranate, black carrot, and red beet juices may contribute to improving antioxidative properties and improve the textural quality of chicken breast meat.

## Graphical abstract

Textural properties increased with marinating processing with fruit and vegetable juices marination. Marinating chicken breast meat with fruit and vegetable juices enhances its technological and physicochemical quality. The sensory properties of the marinated samples provided improved results.

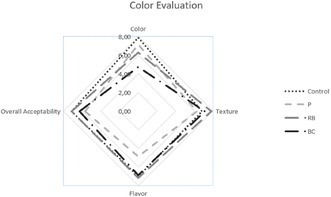

## 1 Introduction

There is an important relationship between health and nutrition. Consumers prefer healthy foods, and it is also important that the food source is economical (U‐Chupaj et al. 2021). Chicken meat constitutes a significant part of the total world meat consumption after pork, as it is an economically easily available and healthy food source with high protein content (Erge et al. 2018). Since chicken is the cheapest commercially produced meat, its consumption is increasing day by day (Kim et al. 2020; Sujiwo et al. 2018). Chicken meat, which is white meat, is preferred for its health, lower fat and cholesterol content, and ease of preparation (Lamri et al. 2021).

Marination is a practice that increases the aroma, flavor, succulence, and tenderness of meat. It also improves the appearance, quality, and shelf life of meat. Marination is one of the methods used to increase chicken meat palatability (Kaewthong and Wattanachant 2018). Marinating is the process of immersing meat in various marinades containing different ingredients for a certain period of time before cooking (Ehsanur Rahman et al. 2023). Marinating extends the appearance, quality, yield, and shelf life of meat. The quality of marinated products can be affected by these factors (Latoch et al. 2023).

Antioxidants are substances that reduce or delay oxidation to a substrate. Antioxidants found in fruits and vegetables help reduce the risk of chronic diseases such as cardiovascular diseases, diabetes, and cancer and protect the health of consumers (Fan et al. 2014). Antioxidants neutralize free radicals, inhibit lipid oxidation, and thus preserve the flavor, aroma, and nutritional value of meat. Preventing oxidative spoilage extends the shelf life of meat and meat products, reducing economic losses (Rivera Toapanta et al. 2022; Vlaicu et al. 2022). Antioxidants preserve the color and texture of meat, increasing consumer acceptance (Unal et al. 2022). Natural antioxidants offer potential health benefits and meet consumer demand for natural products. In recent years, research on the effects of antioxidants on food quality by using antioxidant substances in meat and meat products has gained importance (Yerlikaya and Şen Arslan 2021; Şen Arslan and Yerlikaya 2023; Arı et al. 2023).

Violeta Nour (2022) reported that cherry and plum juices enhance the sensory properties and storage stability of pork fillet, while Augustyńska‐Prejsnar et al. (2023) found that apple and lemon juice improve the microbiological safety and technological properties of marinated poultry meat, and Ilkin Yucel Sengun (2019) demonstrated that verjuice ( *Vitis vinifera* L.) can be used as an alternative marinade to enhance the safety and quality of poultry meat. This study aims to fill a significant gap in the literature, as there is limited research on the use of pomegranate, red beet, and black carrot juices for marinating chicken breast meat. The primary objective is to investigate the effects of these fruit and vegetable juices on lipid oxidation, evaluating their potential as natural antioxidants to enhance meat quality and extend sensory properties. The findings of this study are expected to contribute to the scientific knowledge on the use of natural ingredients as alternatives to synthetic antioxidants in meat products, providing valuable insights for the food industry.

## 2 Material and Methods

### 2.1 Materials

Chicken breast fillets were obtained from eight broilers, each approximately 5 weeks old, from a local market in Konya, Türkiye. Additionally, pomegranate, red beet, and black carrot were purchased from the same market in Ankara, Türkiye. ABTS (2,2′‐azino‐bis(3‐ethylbenzothiazoline‐6‐sulfonic acid)), DPPH (2,2‐diphenyl‐1‐picrylhydrazyl), TBARS (Thiobarbituric Acid Reactive Substances), and Folin (Folin–Ciocalteu reagent) were obtained from Sigma Chemical Co. (St. Louis, MO).

Chicken breast meat used in the research had a pH value of 5.63, and L*, a*, and b* values were measured as 52.58, 1.44, and 6.05, respectively.

### 2.2 Sample Preparation and Marination

In order to prepare the chicken for marination, all external fat and skin were removed from the chicken breasts. The meat was then sliced perpendicularly to the muscle fibers into pieces measuring 2 × 3 × 4 cm. Freshly squeezed juices were obtained from pomegranates, red beets, and black carrots. Approximately 150 g of chicken fillets were placed in separate polypropylene containers (15 cm deep, 35 cm wide). Four different marinades were used: (i) a control group with distilled water, (ii) 100% pomegranate juice (P), (iii) 100% red beet juice (RB), and (iv) 100% black carrot juice (BC). The marinades were added to the containers with a meat‐to‐marinade ratio of 150:250 (w/v). The marination process was carried out for 24 h at 4°C. After this period, the marinade liquid was removed from the containers prior to further analysis.

For color and texture analyses, the samples were cooked using the sous vide method until they reached an internal temperature of 76°C, then cooled before analysis.

### 2.3 Determination of pH and Titratable Acidity

The pH and the titratable acidity were determined according to the method of Porto et al. (2018). The pH of the marinades was determined using an immersion pH meter (WTW Profiline, pH 3110, Germany), while the pH of the marinated meat was measured with a penetration pH meter (Testo 205, Germany). The pH meter has a measurement accuracy of ±0.01 pH units, ensuring precise readings. The instrument was calibrated prior to each measurement session using standard buffer solutions at pH 4.00, pH 7.00, and pH 10.00 to maintain accuracy and reliability.

### 2.4 Determination of TPC, DPPH, ABTS, and TBARS Values

Total phenolic contents (TPC) of the extracts were determined using the method of Singleton et al. (1998). A 2 mL of 10‐fold diluted Folin–Ciocalteu's phenol reagent was mixed with 0.4 mL diluted extract or gallic acid solution (20–100 mg/L). A 1.6 mL sodium carbonate solution (7.5%, w/v) was added to the above mixture. After incubation at room temperature for 1 h, the absorbance was read at 765 nm using a spectrophotometer (Model UV‐1700, Shimadzu Corp., Kyoto, Japan) and the results were expressed as mg gallic acid equivalent (GAE)/L.

DPPH assay was determined according to the method of Singh et al. (2002). Briefly, a 0.1 mL of diluted extract solution was mixed with 3.9 mL of a 25 mg/L methanolic solution of DPPH, and this mixture was vortexed for 10 s. After 30 min of incubation at room temperature, absorbance was measured at 515 nm using a UV–Vis spectrophotometer (Agilent 8453, USA) versus the prepared blank (methanol). The results were expressed as EC_50_ (mg TEAC/mL).

The antioxidant capacity was determined by another radical called ABTS. The solutions of ABTS (2.45 mM) and K_2_S_2_O_8_ (12.25 mM) were mixed and incubated in darkness for 16 h to obtain a stock solution of ABTS radical. The absorbance of the ABTS solution was adjusted to 0.700 ± 0.005 at 734 nm just before the application of the assay. Extracts (20–80 μL) and ABTS solution (2 mL) were mixed and incubated in a darkness for 6 min. The absorbances were measured by a spectrophotometer at 734 nm. The results were expressed as EC_50_ (mg TEAC/mL).

Ten grams of homogenized chicken breast meats (treated and non‐treated), 97.5 mL distilled water, and 2.5 mL 4 N HCl were separately added to the flask and then heated. 5 mL distillate was taken and added to test tubes. All test tubes were heated in hot water at 70°C for 30 min. When color changes in the tubes were seen, absorbance values of all samples were measured at 530 nm using a spectrophotometer. The thiobarbituric acid (TBA) values in chicken breast meats were determined as described above by Alparslan et al. (2016) to evaluate the oxidative stability. The TBA results were expressed as mg of malondialdehyde per kg of chicken meat samples.

### 2.5 Color Measurements

For color measurements, L* (lightness = 0 vs. brightness = 100), a* (redness vs. greenness) and b* (yellowness vs. blueness) values of the cooked and uncooked samples after the marination process were specified using a chromameter CR‐400 (Konica Minolta Inc., Osaka, Japan). The measurements were executed by directly reading on three different parts of the samples (Hunt et al. 1991).

### 2.6 Physicochemical Properties

#### 2.6.1 Determination of Marinade Absorption and Yield

Marinade absorption (MA) and yield were determined following the method outlined by Petracci et al. (2012) by monitoring the weight changes that occurred during both marination and cooking.

#### 2.6.2 Determination of Water Holding Capacity

The water holding capacity (WHC) of the marinated chicken was assessed using an adapted version of the method suggested by Gómez‐Guillén et al. (2000).

#### 2.6.3 Determination of Cooking Loss

The cooking loss (CL) of the marinated meat samples was measured following the procedure outlined by Kondaiah et al. (1985).

### 2.7 Determination of Textural Parameters

For texture profile analysis, marinated chicken meat samples were cooked in a convection oven at 200°C for 40 min, until the internal temperature reached 76°C, following a modified method of Saha et al. (2009). After cooking, the samples were cooled to room temperature (20°C) for further analysis. The textural properties, including hardness, springiness, cohesiveness, chewiness, and resilience, were measured using a texture profile analyzer (TA‐HD Plus Texture Analyzer, UK) equipped with a 50 kg load cell. The meat samples, cut into cuboid shapes (3 × 3 × 1 cm), were compressed to 50% of their original height using a cylindrical probe with a 36 mm diameter (SMS P/36, TA.XT Plus Stable Micro Systems Ltd., Surrey, England) at a speed of 1 mm/min before the test and 5 mm/min during and after the test. The texture profile analysis (TPA) was conducted at room temperature using specialized texture analyzer software (Modi et al. 2009).

### 2.8 Sensory Analysis

Chicken breast meat samples were analyzed by sensory evaluation via a semi‐trained panel of 37 participants, including students and faculty lecturers from the Selcuk University Faculty of Agriculture. Sensory analysis of the chicken breast meat samples was carried out on the first day after marination. For this purpose, chicken breast meats were cooked using an electric grill at 180°C for 15 min, and three‐digit numbers were assigned to each sample randomly. The samples were evaluated for color, texture, flavor, and overall acceptability using a 9‐point scale (9 represented extremely desirable and 1 was extremely undesirable) according to the method given by Modzelewska‐Kapituła et al. (2022). This sensory analysis in this study was reviewed and approved by the Ethics Committee of Selcuk University (approval number: 2025/E958952).

### 2.9 Statistical Analysis

The collected findings were evaluated with Minitab 16 software (Minitab Inc., State College, PA, USA) employing ANOVA at a 95% confidence level (*p* < 0.05). The results were presented as mean ± SD. All analyses were conducted in two repetitions and three parallels (six data) and then analyzed using the Minitab 16 software (Minitab Inc., State College, PA, USA). Statistical analyses of observed differences among means consisted of analysis of variance (ANOVA), followed by Tukey's pairwise comparison of means, with a level of significance defined at 5%.

## 3 Results and Discussion

### 3.1 Physical Properties of Marinades

The pH values of pomegranate (P), red beet (RB), and black carrot (BC) were 3.19, 6.14, and 6.24, respectively, while their titratable acidities were 13.91%, 5.17%, and 3.48% citric acid (Table 1). Among the marinades, P had the lowest pH and the highest titration acidity. Among both cooked and raw samples, P had the highest L* value and the lowest a* value. Meanwhile, BC exhibited the lowest b* value, while RB had the highest a* value.

**TABLE 1:** Some physical and chemical properties of pomegranate, red beet, and black carrot juices.

Analyses	Samples			*p*
	P	RB	BC	
TPC (mg GAE/L)	525.01^c^ ± 45.66	932.23^b^ ± 12.50	1746.07^a^ ± 31.39	0.023
ABTS‐EC_50_ (mg TE/mL)	3.79^a^ ± 0.31	2.51^b^ ± 0.93	0.61^c^ ± 0.22	0.002
DPPH‐EC_50_ (mg TE/mL)	49.68^a^ ± 2.64	41.92^b^ ± 0.91	41.34^b^ ± 0.09	0.012
pH	3.19^c^ ± 0.01	6.14^b^ ± 0.01	6.24^a^ ± 0.02	0.003
L*	42.61^a^ ± 1.05	11.51^b^ ± 0.54	12.14^b^ ± 0.79	0.005
a*	40.62^a^ ± 1.04	14.78^b^ ± 0.62	15.51^b^ ± 0.45	0.024
b*	19.12^a^ ± 0.25	−2.77^b^ ± 0.35	−4.51^c^ ± 0.57	0.003
Titratable acidity (%citric acid percentage)	13.91^a^ ± 0.15	5.17^b^ ± 0.34	3.48^c^ ± 0,32	0.005

*Note:* Values represent the mean ± standard deviation. Different superscript letters within the same row are significantly different (*p* < 0.05) by Tukey test.

Abbreviations: BC, black carrot juice; P, pomegranate juice; RB, red beet juice.

Violeta Nour (2022) observed low pH levels and high titration acidity in pork loin marinated with cherry and plum juices. It is reported that the acidic environment alters the protein structure of the meat and therefore may increase the penetration of the marinade and affect color and textural properties. Augustyńska‐Prejsnar et al. (2023) reported that the pH level decreased in poultry meat marinated with apple and lemon juice, and this had a positive effect on microbial stability. In our study, BC had the lowest b* value, which supports the effect of vegetable juices with high polyphenol content on color changes. In addition, the fact that RB had the highest a* value can be explained by the betalain pigments in red beet and is consistent with other studies. These comparisons show that the effect of different fruit and vegetable juices on the chemical and color properties of meat is consistent with studies in the literature and that the ingredients used in the marinating process play an important role in shaping the quality of meat.

### 3.2 TPC and DPPH, ABTS, TBARS

Polyphenols are bioactive compounds that occur naturally in plant‐derived foods such as fruits and vegetables (Liu et al. 2023). TPC of juices is expressed in Table 1. The TPC of the juices ranged from 525.01 to 1746.07 mg GAE/L. There was a significant difference (*p* < 0.05) between the TPC of juices. The highest TPC was found in BC. The results of antioxidant activity of juices were expressed using the term EC_50_ (Table 1). The lower the EC_50_, the higher the antioxidant activity. If a comparison was made in terms of antioxidant activity, BC juice had higher antioxidant activity than P and RB juices (*p* < 0.05).

Lipid oxidation occurs when pro‐oxidant factors pass the antioxidant compounds and factors added to meat products (Alirezalu et al. 2019). The TBARS assays indicate the level of the secondary products produced by lipid oxidation (Lorenzo et al. 2018). Due to the high antioxidant content of the marinades used in the study, there was a statistically significant difference (*p* < 0.05) between them after marination. TBARS values of marinated samples were found to be lower compared to the control sample. The use of these antioxidant marinades can be effective in delaying oxidation.

**FIGURE 1:**
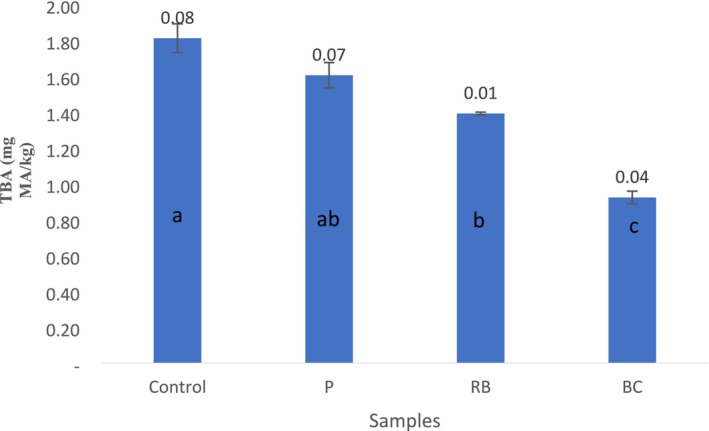
Effects of red beet, black carrot, and pomegranate juices on the TBARS (mg of MDA/kg) values of chicken breast samples. (Values represent the mean ± standard deviation. BC, black carrot juice; P, pomegranate juice; RB, red beet juice. Different superscript letters within the same row are significantly different (*p* < 0.05) by Tukey test.)

It was determined that the effect of marination on TBARS results was statistically important (*p* < 0.05) (Figure 1). The highest TBARS level (1.81 ± 0.08 mg malonaldehyde/kg sample) belonged to the control sample (without marinade). Marination processes decreased the TBARS level. The minimum TBARS level (0.92 ± 0.04 mg malonaldehyde/kg sample) belonged to samples marinated with BC.

According to Figure 1, it has been observed that the high TPC and antioxidant activity of fruit juices reduces TBARS.

These substances have many biological functions, mainly including antioxidant activity. The marinades used in this study are very rich in TPC. For this reason, the low TBARS values of marinated samples can be explained by the fact that marinades contain high amounts of phenolics and have antioxidant activity. Studies have found that TBARS values decrease when meat and meat products are treated with substances rich in phenolic compounds (Şen Arslan and Yerlikaya 2023; Arı et al. 2023; Yerlikaya and Şen Arslan 2021; Oğuz et al. 2019).

### 3.3 Color Measurements

Color is crucial for the attractiveness of meat and meat products (Nour 2022). Color is a characteristic that influences consumer preference and plays an important role in the quality of meat and meat products (Xu et al. 2018). Table 2 shows color values (L*, a*, and b*) of raw and cooked chicken breast meats treated with different marinades (control, black carrot juice [BC], pomegranate juice [P] and red beet juice [RB]).

**TABLE 2:** Color parameters values of raw and cooked chicken breast meats treated with different marinades.

Type	Parameters	Control	BC	P	RB	*p*
Uncooked	L*	63.38 ± 1.39^a^	24.05 ± 1.60^c^	52.93 ± 1.70^b^	16.08 ± 0.89^d^	0.002
	a*	2.56 ± 0.26^d^	16.18 ± 0.63^b^	7.50 ± 0.87^c^	27.38 ± 1.07^a^	0.000
	b*	6.83 ± 0.64^a^	0.64 ± 0.22^d^	5.58 ± 0.76^b^	3.18 ± 0.52^c^	0.015
Cooked	L*	75.26 ± 0.62^a^	35.33 ± 0.96^c^	57.90 ± 0.89^b^	35.01 ± 0.79^c^	0.042
	a*	1.60 ± 0.21^d^	10.14 ± 0.61^b^	4.01 ± 0.43^c^	24.84 ± 0.62^a^	0.026
	b*	15.10 ± 0.65^a^	0.86 ± 0.62^b^	15.23 ± 0.70^a^	15.93 ± 0.90^a^	0.009

*Note:* Values represent the mean ± standard deviation. Different superscript letters within the same row are significantly different (*p* < 0.05) by Tukey test.

Abbreviations: BC, black carrot juice; RB, red beet juice.

In this study, immersing in fruit juice marinades had a significant effect on the color values of both uncooked and cooked chicken breast meat samples (*p* < 0.05). The L* value, an indicator of lightness, was lower in the marinated chicken breast meat compared to the control group. The presence of anthocyanins in the fruit juice used is thought to be responsible for the lower L* value in the marinated samples. These findings are consistent with the data from Nour's (2022) study, which examined the effects of sour cherry juice marinades on pork loin. The uncooked and cooked control group displayed the highest L* values (lightness = 0 vs. brightness = 100) (63.38 and 75.26, respectively) among the other groups. The lowest L* value (16.08 and 35.01, respectively) was measured in the samples treated with red beet juice.

It was observed that marinating in fruit juice contributed to the a* value (redness vs. greenness) of all the samples. The chicken sample treated with red beet juice had the highest a* value (27.38 and 24.84 for cooked and uncooked samples). Additionally, treating with black carrot juice decreased the b* values (yellowness vs. blueness) (0.64 and 0.86 for cooked and uncooked samples) of the samples. However, when the cooked groups were examined, no significant difference (*p* > 0.05) was found in terms of b* parameters in the chicken breast meat samples except for the sample treated with black carrot juice.

In a study by Unal et al. (2020), a similar L* value (51.21) was observed in chicken meat marinated with pomegranate juice. Our data aligns with the findings of Gök and Bor (2016), who investigated the effects of pomegranate and black carrot juice on turkey meat. In that study, the highest lightness value was found in the control samples for both cooked and uncooked groups, similar to our results. Conversely, Augustyńska‐Prejsnar et al. (2023) reported that using whey and lemon juice as marinades lightened the color of raw breast muscles compared to non‐marinated ones, attributing this to the marinades' lower pH and the increased extracellular water introduced into the meat. Beltrán‐Cotta et al. (2023) observed that the lightness value of Boston butt pork increased when yellow mombin was used at a 100% concentration. This was attributed to the acidity of yellow mombin, which causes protein denaturation and alters light reflectance and ionic strength.

### 3.4 Physicochemical Properties

The pH values of chicken meat samples varied between 4.21 and 5.96 (Table 3). Marination with P, RB, and BC decreased the pH of the meat. The reason for this was thought to be the presence of organic acids in the marinades. The lowest pH was observed in P‐marinated chicken meat with the highest titratable acidity, while the highest pH was determined in the control sample. Unal et al. (2022) reported that marinating with lemon juice decreased pH. Similar results were reported in studies in the literature where turkey breast meat (Gök and Bor 2016), spent hen meat (Dilek et al. 2023) and beef (Unal et al. 2023) were marinated with various fruit and vegetable juices and vinegars.

**TABLE 3:** Effects of red beet, black carrot, and pomegranate juices on the pH, MA, WHC, CL, and yield parameters of the chicken breast samples.

Parameters	Control	P	RB	BC	*p*
pH	5.96 ± 0.163^a^	4.21 ± 0.120^c^	5.62 ± 0.014^ab^	5.53 ± 0.007^b^	0.000
MA	2.46 ± 0.182^b^	−3.11 ± 0.031^c^	3.71 ± 0.020^ab^	4.45 ± 0.654^a^	0.000
WHC (%)	22.17 ± 0.192^a^	23.44 ± 0.822^a^	25.53 ± 1.864^a^	24.75 ± 1.687^a^	0.199
CL (%)	25.89 ± 2.559^a^	20.22 ± 1.047^b^	18.98 ± 0.019^b^	16.97 ± 0.098^b^	0.012
Yield (%)	58.46 ± 1.396^b^	62.92 ± 0.399^a^	64.15 ± 0.433^a^	65.27 ± 1.218^a^	0.008

*Note:* Values represent the mean ± standard deviation. Different superscript letters within the same row are significantly different (*p* < 0.05) by Tukey test.

Abbreviations: BC, black carrot juice; RB, red beet juice.

The marinade absorption (MA) is used to assess the quality of chicken meat. During the marination process, spoilage is inhibited by lowering the water activity, and a distinct flavor is infused into the meat (Li et al. 2020). MA values were in the range of −3.11% to 4.45%, and marinades except P caused a significant increase (*p* < 0.05) in MA compared to the control group (Table 3). Since P marinade has higher acidity than the other groups, it may denature the proteins and cause a decrease in their functional properties, such as water binding. In the study of Dilek et al. (2023), MA was lower in all groups compared to the control. In the study of Unal et al. (2022), MA was found to be lower in citric acid and grapefruit juice treatments and higher in the lemon juice sample compared to the control group. In another study, it was reported that marination with various vinegars increased MA compared to the control group (Unal et al. 2023). This difference in the literature results may be due to different meat types, the growing and slaughtering conditions of the animals from which the meat originated, or differences in marinades (concentration, pH, density, etc.).

According to Table 3, water holding capacity (WHC) values of chicken meat marinated with P, RB, and BC were determined in the range of 22.17%–25.53%. The lowest value in this range was in the control group, and the highest value was in the RB sample. In parallel with these results, in a marination study with citric acid and acidic fruits, it was reported that the WHC of all samples was higher than that of the control group. WHC is impacted by marination with fruit and vegetable juices, largely due to changes in the structural characteristics of muscle fibers. Acidic ingredients lower the pH of the meat, causing the protein network to expand, which helps the meat retain more water. Additionally, the presence of dietary fibers in fruits and vegetables used in the marinade enhances WHC, as these fibers tend to bind water effectively, forming a more complex network within the meat (Latoch et al. 2023).

Cooking loss (CL) values of chicken meat marinated with P, RB, and BC are given in Table 3. The CL values ranged from 16.97% to 25.89%. Marinating with various fruit and vegetable juices significantly decreased the CL values compared to the control (*p* < 0.05). The lowest CL value was observed in chicken meat marinated with BC, while the control sample had the highest CL value. In a study in which turkey meat was marinated with various fruit and vegetable juices, including black carrot and pomegranate, it was reported that the CL of samples with BC and P was lower than the control group in 24 h of marination (Gök and Bor 2016). Similar results were found in the study carried out by Unal et al. (2023) and Dilek et al. (2023). Acidic components in marinades lower the pH of meat and bring it closer to the isoelectric point pH. However, the acidic effect leads to the denaturation of proteins. For these reasons, water may be better retained in muscle fibers, resulting in less water loss when meat is cooked.

A significant increase was observed in yield values with different marinades applied to chicken meat compared to the control group (*p* < 0.05). While the control group had the lowest yield among the marinated chicken meat, the highest yield was observed in the sample marinated with BC. In a marination study with various vinegars, it was reported that the group with hawthorn vinegar (73.79%) increased the yield compared to the control (Dilek et al. 2023). In another study using fruit vinegars, it was reported that the treatment groups did not increase the yield compared to the control (Unal et al. 2023). It is seen in Table 3 that the yield in all groups is directly proportional to WHC and inversely proportional to CL.

### 3.5 Texture Properties of Marinated Chicken Meat

Texture profile results of chicken meat marinated with P, RB, and BC are given in Table 4. Hardness values were significantly affected by marinating chicken meat (*p* < 0.05). Hardness values ranged from 135.23 to 209.52 N, with the sample marinated in black carrot juice exhibiting the lowest hardness value compared to the control group. However, higher hardness values were obtained as a result of marination with pomegranate and red beetroot juices compared to the control group. This is thought to be due to the higher acidic properties of pomegranate compared to other marinades. The high acidity of pomegranate juice may have increased the hardness of chicken breast meat by denaturing meat proteins and binding protein fibers more densely. Similarly, a study by Unal et al. (2022) that marinated chicken breast meat with citric acid, grapefruit, and lemon juices found that the marinade containing citric acid increased the hardness value compared to the control. In another study by Gök and Bor (2016), where turkey breast meat was marinated with different fruit and vegetable juices, it was reported that the marinade containing pomegranate juice increased the hardness value in cooked turkey breast meat, and this group also had the lowest pH value.

**TABLE 4:** Effects of red beet, black carrot, and pomegranate juices on the textural properties of the cooked chicken breast samples.

Parameters	Control	P	RB	BC	*p*
Hardness (*N*)	178.53 ± 6.011^b^	209.52 ± 6.423^a^	206.67 ± 2.490^a^	135.23 ± 5.212^c^	0.000
Springiness (mm)	0.60 ± 0.016^b^	0.69 ± 0.037^ab^	0.70 ± 0.058^ab^	0.77 ± 0.044^a^	0.063
Cohesiveness (dimensionless)	0.55 ± 0.001^a^	0.53 ± 0.005^a^	0.52 ± 0.036^a^	0.55 ± 0.039^a^	0.592
Chewiness (*N*)	58.57 ± 0.624^a^	76.22 ± 1.157^a^	74.70 ± 10.468^a^	56.99 ± 5.189^a^	0.057
Resilience (dimensionless)	0.17 ± 0.006^a^	0.18 ± 0.001^a^	0.15 ± 0.012^a^	0.18 ± 0.017^a^	0.072

*Note:* Values represent the mean ± standard deviation. Different superscript letters within the same row are significantly different (*p* < 0.05) by Tukey test.

Abbreviations: BC, black carrot juice; RB, red beet juice.

Springiness parameter of chicken breast samples was significantly affected by marinating with different fruit and vegetable juices (*p* < 0.05). Springiness refers to the ability of a food to return to its original shape after compression. As seen in Table 4, the lowest springiness value belongs to the control group (0.60 mm), and the highest value is in the BC group (0.77 mm). This proves that the BC group has a more elastic structure. Dilek et al. (2023) marinated chicken breast meat with aronia, hawthorn, and grape vinegars and found similar results in terms of the springiness parameter. BC had the lowest value in the chewiness parameter. This indicates that the meat can be consumed with less chewing than the other groups and is an important parameter in terms of consumer acceptance. In summary, since the pH of BC was higher than the isoelectric point pH of myofibrillar proteins, it caused marinade absorption and less cooking loss in the meat. Thus, the muscle fibers became looser, more flexible, and juicier in the BC marinated group compared to the other treatment groups. This resulted in a softer and more easily chewed chicken breast meat.

### 3.6 Sensory Evaluation

**FIGURE 2:**
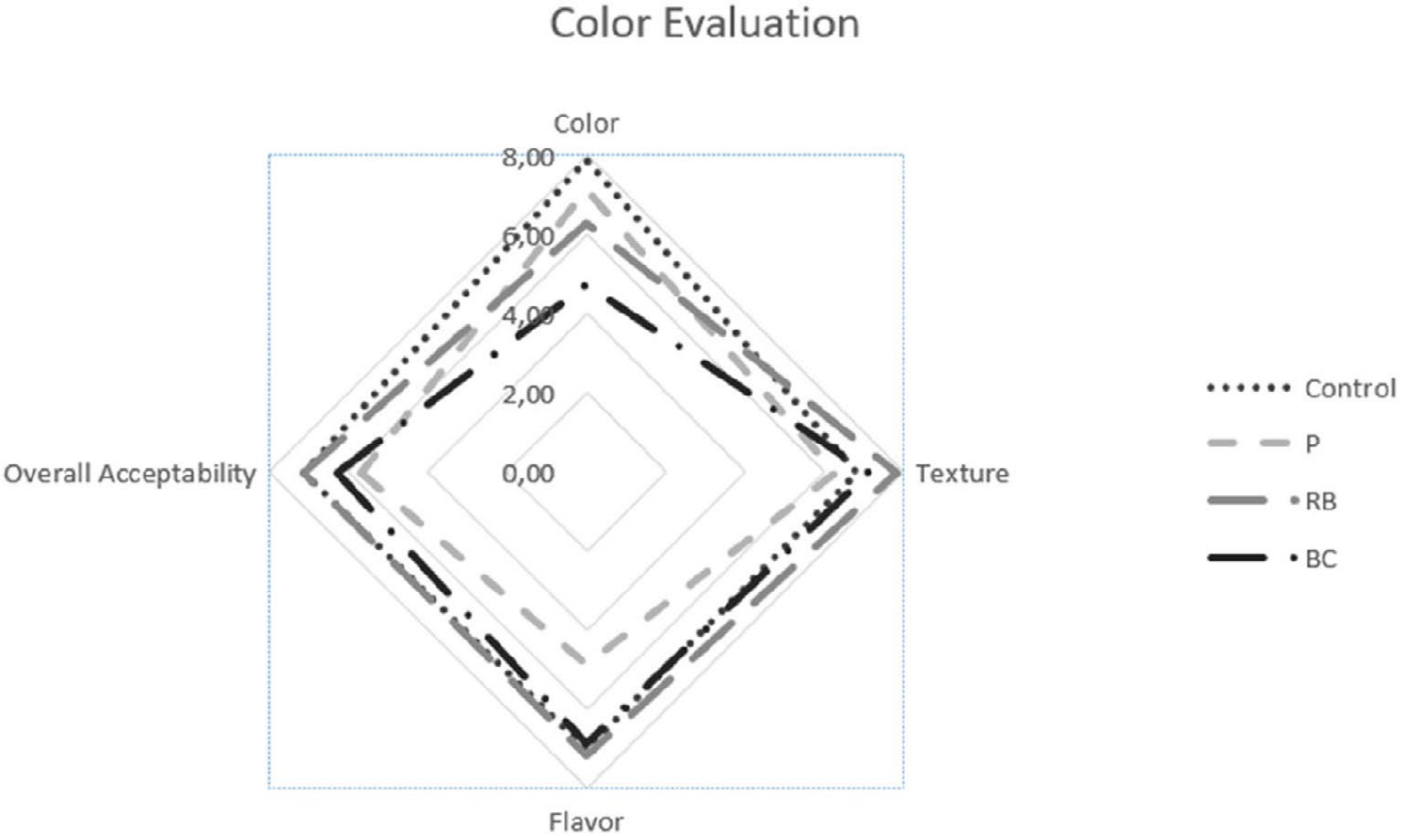
Sensory scores of the chicken breast meats treated with different marinades.

Figure 2 illustrates the color, texture, flavor, and overall acceptability ratings of the marinated chicken breast meat samples. The data provide a comprehensive comparison of how different marinades influenced the sensory attributes of the chicken, highlighting variations in visual appeal, mouthfeel, taste, and overall consumer preference. These insights are crucial for understanding the impact of marination on the quality and palatability of the meat.

Any statistically significant differences were not observed among the chicken breast samples with respect to the texture and flavor (*p* > 0.05); however, the color parameter was affected by marination (*p* < 0.05). When overall acceptability was observed, it was seen that marinating chicken breast meat with fruit juices did not cause negative changes in consumer perception. These results were in line with the results of research on the effects of yellow mombin on Boston butt pork (Beltrán‐Cotta et al. 2023).

According to the sensory evaluation results, BC had the lowest score for color, whereas the highest color score was given to the control. The texture sensory scores had close values and were not significantly different for all the groups. Although the highest score belonged to P in terms of flavor, the overall acceptability results showed this group was less desirable in comparison with the other groups. Control and RB had the same score in terms of overall acceptability (*p* > 0.05). Unal et al. (2020) reported that all samples were statistically equally acceptable, indicating that the marinades did not significantly alter overall consumer preference. Similarly, Gök and Bor (2016) observed comparable results in the general acceptability of turkey breast meat, finding that the samples marinated in black carrot juice and pomegranate juice received higher scores than the control group. These findings suggest that certain fruit and vegetable juices can enhance the palatability of poultry products without compromising consumer acceptance.

## 4 Conclusions

In this study, marinating chicken breast meat with fruit juices resulted in a decrease in pH due to the presence of organic acids in the marinades. The lightness parameter (L*) was lower in the marinated samples compared to the control group, likely attributed to the anthocyanins present in the fruit juices. Low TBARS values, which are an indicator of antioxidant activity, show that oxidation is better controlled and the quality of the meat is preserved. The TBARS values of the marinated samples were found to be lower than those of the control sample, suggesting that these antioxidant marinades can effectively delay oxidation. In this context, BC marinade provided the lowest TBARS values and showed the most effective result. In marination, the most important parameter in terms of textural aspects is hardness, and it is seen that the lower the hardness value, the more successfully the purpose of the marination is achieved. As a result of the analyses, it was determined that the best hardness value was obtained with BC. The lowest and highest water holding capacity (WHC) values were observed in the control group and the red beet marinated group, respectively. In marination, the most important parameter in terms of textural hardness is hardness, and it is seen that the lower the hardness value, the more successfully the purpose of marination is achieved. These variations may be attributed to changes in the structural characteristics of the muscle fibers. Hardness values were significantly influenced by marination (*p* < 0.05). As a result of the analyses, it was determined that the best hardness value was obtained with BC. Sensory evaluation results indicated that the black carrot (BC) received the lowest score for color, while the highest color score was awarded to the control group. Both the control and red beet (RB) received the same score for overall acceptability. In conclusion, marinating chicken breast meat with red beet, pomegranate, and black carrot juices can enhance its textural, technological, and physicochemical quality.

## Author Contributions

**İlkay Çelik:** conceptualization (equal), investigation (equal), writing – review and editing (equal). **Eda Alagöz:** formal analysis (equal), investigation (equal), writing – review and editing (equal). **Hülya Şen Arslan:** methodology (equal), visualization (equal), writing – original draft (lead). **Cemalettin Sarıçoban:** funding acquisition (equal), supervision (equal).

## Disclosure

The authors have nothing to report.

## Data Availability

All data generated or analyzed during this study are included in this published article.
